# Multivariate models of brain volume for identification of children and adolescents with fetal alcohol spectrum disorder

**DOI:** 10.1002/hbm.24867

**Published:** 2019-11-18

**Authors:** Graham Little, Christian Beaulieu

**Affiliations:** ^1^ Department of Biomedical Engineering University of Alberta Edmonton Alberta Canada

**Keywords:** classification, fetal alcohol spectrum disorder (FASD), machine learning, neurodevelopment

## Abstract

Magnetic resonance imaging (MRI) studies of fetal alcohol spectrum disorder (FASD) have shown reductions of brain volume associated with prenatal exposure to alcohol. Previous studies consider regional brain volumes independently but ignore potential relationships across numerous structures. This study aims to (a) identify a multivariate model based on regional brain volume that discriminates children/adolescents with FASD versus healthy controls, and (b) determine if FASD classification performance can be increased by building classification models separately for each sex. Three‐dimensional T1‐weighted MRI from two independent childhood/adolescent datasets were used for training (79 FASD, aged 5.7–18.9 years, 35 males; 81 controls, aged 5.8–18.5 years, 32 males) and testing (67 FASD, aged 6.0–19.6 years, 38 males; 74 controls, aged 5.2–19.5 years, 42 males) a classification model. Using FreeSurfer, 87 regional brain volumes were extracted for each subject and were used as input into a support vector machine generating a classification model from the training data. The model performed moderately well on the test data with accuracy 77%, sensitivity 64%, and specificity 88%. Regions that contributed heavily to prediction in this model included temporal lobe and subcortical gray matter. Further investigation of two separate models for males and females showed slightly decreased accuracy compared to the model including all subjects (male accuracy 70%; female accuracy 67%), but had different regional contributions suggesting sex differences. This work demonstrates the potential of multivariate analysis of brain volumes for discriminating children/adolescents with FASD and provides indication of the most affected regions.

## 1 INTRODUCTION

A diagnosis of fetal alcohol spectrum disorder (FASD) relies on the identification of physical, cognitive, and behavioral impairments related to prenatal alcohol exposure (PAE; Popova et al., 2016). Quantitative structural magnetic resonance imaging (MRI) studies have consistently reported reductions of total brain, white matter, and gray matter volumes in individuals with prenatal exposure to alcohol who are often diagnosed with FASD (for reviews, see Donald et al., 2015; Lebel, Roussotte, & Sowell, 2011). Some structures may be disproportionately affected in FASD with larger proportional reductions in specific deep gray matter structures such as the caudate and putamen (Nardelli, Lebel, Rasmussen, Andrew, & Beaulieu, 2011; Roussotte et al., 2012). These brain volume reductions have also been reported in infants and neonates with PAE for the corpus callosum (Jacobson et al., 2017) and gray matter (Donald et al., 2016). In addition, larger volume reductions have been observed in males with FASD suggesting sex differences (Chen, Coles, Lynch, & Hu, 2012; Dudek, Skocic, Sheard, & Rovet, 2014; Treit et al., 2017). However, most of these studies analyze each brain region separately (i.e., univariate analysis) and volumes have considerable overlap between groups making them unsuitable for individual FASD diagnosis.

Machine learning classification takes multiple variables as input to build a multivariate classification model capable of separating groups based on the provided input. In short, a multivariate classification model is a mathematical equation that describes a multidimensional boundary (e.g., a plane) where data points located on opposite sides of the boundary are classified into different groups (i.e., FASD vs. control). Machine learning classification of neuroimaging features has shown promise to discriminate individuals with brain disorders from healthy controls (Arbabshirani, Plis, Sui, & Calhoun, 2017). These techniques have been applied in pediatric populations to identify neurodevelopment disorders such as attention deficit hyperactivity disorder (ADHD) and autism (Levman & Takahashi, 2015). Multivariate classification studies with neuroimaging data typically rely on a large number of samples to achieve stable models (Nieuwenhuis et al., 2012) and to date ADHD classification studies have been performed most often on the same cohort of children and adolescents collected as part of the ADHD‐200 consortium (Milham, Fair, Mennes, & Mostofsky, 2012). Classification models on the ADHD‐200 data have achieved accuracies ranging from 55% using structural brain features (Colby et al., 2012) to 81% using resting‐state functional connectivity features (Fair et al., 2013) in classifying children/adolescents with ADHD. Similar accuracies have been achieved in studies of large cohorts (>100 participants) of children/adolescents with autism reporting classification accuracies of between 70% using a combination of regional brain volume and functional connectivity features (Zhou, Yu, & Duong, 2014) to 91% using functional connectivity features alone (Chen et al., 2015). To our knowledge, only one study focusing on eye tracking and psychometric data has attempted FASD classification using neuroimaging‐based features. This study extracted features from diffusion MRI of the corpus callosum, and achieved an accuracy of 65–70% in classifying children/adolescents with FASD (41 individuals with FASD, 35 controls) (Zhang et al., 2019) that was a subset of the larger cohort used in the current study. However, to date, no study has investigated the utility of multivariate classification models using regional brain volumes (notably the most consistent finding across FASD MRI studies) in FASD. Additionally, classification studies of neurodevelopmental disorders typically use a linear regression to reduce sex‐related variation of input features; however, in cases where there are group by sex interactions (e.g., those observed in FASD) this would be suboptimal.

This study had two key aims to: (a) identify a multivariate model based on regional brain volume capable of discriminating children/adolescents with FASD and (b) determine if FASD classification performance can be increased by building classification models separately for each sex given the known volume differences between males and females as a group (Cahill, 2006; Cosgrove, Mazure, & Staley, 2007). The brain volume model was developed and then tested on independent FASD/un‐exposed control cohorts from two studies—a four‐site pan‐Canadian “NeuroDevNet” cohort (79 FASD, 81 controls) and a local single‐site “Canadian Institutes of Health Research (CIHR)” cohort (67 FASD, 74 controls).

## 2 METHODS

### 2.1 FASD/typically developing subjects training and testing datasets

Two previously collected independent MRI datasets were used to generate and validate a predictive model. The training data were collected at four different sites across Canada as part of the NeuroDevNet project on FASD (Reynolds et al., 2011) and was selected as the training dataset so that outputted models were generalizable to different centers or scanners. One hundred and eighty‐one childhood/adolescent healthy and FASD participants underwent brain MRI at four sites, but 21 subjects (11 FASD, 10 controls) were excluded for poor structural imaging quality. The remaining 160 subjects included 79 children with FASD (12.7 ± 3.2 years, 35 males) and 81 healthy unexposed controls (11.9 ± 3.4 years, 32 males). Group analysis of brain volumes has been reported elsewhere for the healthy controls and FASD groups in this cohort (Zhou et al., 2017). FASD participants were recruited from six clinics across Canada and had an alcohol‐related disorder in accordance with the Canadian Guidelines for diagnosis of FASD (Chudley et al., 2005) or had confirmed PAE. The FASD participants in the training data included seven fetal alcohol syndrome (FAS), 13 partial FAS (pFAS), 38 alcohol‐related neurodevelopmental disorder (ARND), and 21 confirmed PAE. In this study, subtypes were combined into two diagnostic groups, either 20 FASD with sentinel facial features (FAS or pFAS) or 38 FASD without sentinel facial features (ARND) in‐line with updated diagnostic guidelines (Cook et al., 2016). PAE subjects remained in a single group as the diagnostic guidelines characterize this group as “at risk of neurodevelopmental disorder and FASD.” All FASD subtypes were labeled as a single group for machine learning classification.

The testing data for model validation was collected under a CIHR project on brain development. Participants with brain MRI included 67 participants with FASD (12.1 ± 3.3 years, 38 males) and 74 controls (11.5 ± 3.5 years, 42 males). Notably, 57 FASD and 66 control participants were included in our previous study on volumes/DTI/cortical thickness (Treit et al., 2017). The other 10 FASD participants were included in a much earlier diffusion MRI study (Lebel et al., 2008), and were the participants that did not overlap the FASD participants from (Treit et al., 2017). An additional eight controls were randomly selected males from a typical development cohort (Narvacan, Treit, Camicioli, Martin, & Beaulieu, 2017) and were added to provide a similar ratio of males and females in the control and FASD groups. All three studies combined for the test data used the same three‐dimensional (3D) MPRAGE protocol on the same scanner at the University of Alberta. Participants from the FASD group were recruited primarily through an FASD diagnostic clinic at the Glenrose Rehabilitation Hospital in Edmonton, AB, and were diagnosed based on Canadian guidelines (Chudley et al., 2005) and the 4‐digit diagnostic code (Astley, 2004). The FASD participants in the testing data included 10 FAS, four pFAS, two ARND, one fetal alcohol effect (FAE), seven neurobehavioral disorder alcohol exposed (NBD:AE), nine static encephalopathy alcohol exposed (SE:AE), 16 “FASD” without further specification, and 18 with no FASD diagnosis but confirmed PAE. As in the training data, subtypes were combined into two diagnostic groups, either 14 FASD with sentinel facial features (FAS or pFAS) or 35 FASD without sentinel facial features (ARND, FAE, NBD:AE, SE:AE, or FASD) consistent with updated diagnostic guidelines (Cook et al., 2016). All FASD subtypes were labeled as a single group for the testing of the machine learning classification model. Further demographic information for training and testing datasets was collected via questionnaire including ethnicity and current medication and is summarized for the training and testing cohorts in Tables 1 and 2, respectively.

**Table 1:** Participant characteristics and demographics for training “NeuroDevNet” data

	Control	FASD	*p*‐Value^a^
Participant characteristics	*n* = 81	*n* = 79	
Age (years)	11.9 ± 3.4 (5.8–18.5)	12.7 ± 3.2 (5.7–18.9)	.138
Males (%)	32 (40%)	35 (44%)	.540
FASD subtype (%)			
FASD with sentinel facial features	0 (0%)	20 (25%)	<.001*
FASD without sentinel facial features	0 (0%)	38 (48%)	<.001*
Confirmed PAE	0 (0%)	21 (27%)	<.001*
Ethnicity (%)			
Indigenous	1 (1%)	41 (52%)	<.001*
Caucasian	74 (91%)	24 (30%)	<.001*
Other	5 (6%)	14 (18%)	.024*
Unknown	1 (1%)	0 (0%)	.323
Medication (%)			
Stimulants	1 (1%)	12 (15%)	.001*
Antidepressants	0 (0%)	3 (4%)	.078
Antipsychotics	0 (0%)	3 (4%)	.078
Stimulants and antipsychotics	0 (0%)	8 (10%)	.003*
Stimulants, antipsychotics, and antidepressants	0 (0%)	2 (3%)	.151
Other	7 (9%)	24 (30%)	<.001*
No medication	73 (90%)	38 (48%)	<.001*
Comorbidities (%)			
ADHD	1 (1%)	40 (50%)	<.001*
Anxiety	0 (0%)	10 (13%)	.001*
Depression	0 (0%)	4 (5%)	.041*
Bipolar	0 (0%)	2 (3%)	.151
Oppositional defiant disorder	0 (0%)	6 (8%)	.012*
Conduct disorder	0 (0%)	2 (3%)	.151
Autism	0 (0%)	1 (1%)	.311
Other disorder	0 (0%)	23 (29%)	<.001*
Site (%)			
University of Alberta	42 (52%)	34 (43%)	.266
Queens University	18 (22%)	22 (28%)	.413
University of Manitoba	8 (10%)	10 (13%)	.579
University of British Columbia	13 (16%)	13 (16%)	.945
Cognitive test (age standardized score)			
Woodcock Johnson Quantitative Concepts 18A&B mathematics	*n* = 80/81	*n* = 78/79	
	105 ± 12 (69–129)	83 ± 19 (37–129)	<.001*
WRMT‐R**‐**Word Identification	*n* = 80/81	*n* = 78/79	
	106 ± 13 (71–134)	91 ± 14 (52–126)	<.001*

Abbreviations: CHIR, Canadian Institutes of Health Research; FASD, fetal alcohol spectrum disorder; PAE, prenatal alcohol exposure; WRMT‐R, Woodcock Reading Mastery Tests‐Revised.

a Group differences of categorical variables (e.g., sex) assessed with Mann–Whitney *U* test; continuous variable (e.g., age) assessed with independent samples *t* test (*p < .05).

**Table 2:** Participant characteristics and demographics for testing “CIHR” data

	Control	FASD	*p*‐Value^a^
Participant characteristics	*n* = 74	*n* = 67	
Age (years)	11.5 ± 3.5 (5.2–19.5)	12.1 ± 3.3 (6.0–19.6)	.26
Males (%)	42 (57%)	38 (57%)	.99
FASD subtype (%)			
FASD with sentinel facial features	0 (0%)	14 (21%)	<.001*
FASD without sentinel facial features	0 (0%)	35 (52%)	<.001*
Confirmed PAE	0 (0%)	18 (27%)	<.001*
Ethnicity (%)			
Indigenous	1 (1%)	19 (28%)	<.001*
Caucasian	55 (74%)	15 (22%)	<.001*
Other	8 (11%)	5 (7%)	<.001*
Unknown	10 (14%)	28 (42%)	<.001*
Medication (%)			
Stimulants	0 (0%)	19 (28%)	<.001*
Atypical antipsychotics	0 (0%)	22 (33%)	<.001*
Antidepressants	0 (0%)	10 (15%)	<.001*
Other	0 (0%)	9 (13%)	<.001*
Comorbidities (%)			
ADHD	0 (0%)	33 (49%)	<.001*
Anxiety	2 (3%)	12 (18%)	<.001*
Reactive attachment disorder	0 (0%)	8 (12%)	<.001*
Other disorder	0 (0%)	17 (25%)	<.001*
Cognitive test (age standardized score)			
Woodcock Johnson Quantitative Concepts 18A&B mathematics	*n* = 66/74	*n* = 52/67	
	107 ± 13 (77–135)	82 ± 13 (53–118)	<.001*
WRMT‐R**‐**Word Identification	*n* = 66/74	*n* = 52/67	
	107 ± 13 (81–147)	89 ± 14 (52–134)	<.001*

Abbreviations: CHIR, Canadian Institutes of Health Research; FASD, fetal alcohol spectrum disorder; PAE, prenatal alcohol exposure; WRMT‐R, Woodcock Reading Mastery Tests‐Revised.

a Group differences of categorical variables (e.g., sex) assessed with Mann–Whitney *U* test; continuous variable (e.g., age) assessed with independent samples *t* test (*p < .05).

This study was approved by the Human Research Ethics Boards at Queens's University, University of Alberta, Children's Hospital of Eastern Ontario, University of Manitoba, and the University of British Columbia. Written informed consent was obtained from parent or legal guardian of children/adolescents. Assent was obtained from each child/adolescent before study participation.

### 2.2 Cognitive testing

Cognitive assessments were performed on the day of the MRI scan at all four sites by research assistants trained by the same neuropsychologist for between site consistency. Research assistants were not blinded to FASD diagnosis and participants took their medication as usual on the days of behavioral testing. The cognitive batteries performed for both the training and testing datasets were different but included evaluations of core functions affected in PAE such as math, reading, executive function, memory, and inhibition. For a full summary of the behavioral tests, see previously published work for training data (Zhou et al., 2017) and testing data (Treit et al., 2017). Only behavioral tests that were conducted in the majority of participants in both the training/testing cohorts were included for analysis in the current study: the Woodcock Johnson III Tests of Achievement evaluated mathematic and quantitative reasoning skills (Woodcock, McGrew, & Mather, 2001) and the Woodcock Reading Mastery Tests‐Revised (WRMT‐R) provided a comprehensive assessment of reading ability (Woodcock, 1998). Results for behavioral tests for the participants/cognitive tests in the current study are presented for both the training and testing groups in Tables 1 and 2, respectively.

### 2.3 Image acquisition

The training “NeuroDevNet” MRI data were acquired at four MR imaging centers: University of Alberta (1.5 T Siemens Sonata), Queen's University (3 T Siemens Trio), University of Manitoba (3 T Siemens Trio), and University of British Columbia (3 T Philips Intera). 3D T1‐weighted images were acquired with high‐resolution (1 × 1 × 1 mm^3^) MPRAGE using 160 axial slices, TI = 1,100 ms, and flip angle = 15°, but repetition (TR) and spin echo (TE) times were set individually per site given variations in scanner performance resulting in slightly different acquisition times: University of Alberta—TE = 4.38 ms, TR = 2,180 ms, scan time 5:41 min; Queens University—TE = 3.45 ms, TR = 2,180 ms, scan time 5:15 min; University of Manitoba—TE = 3.45 ms, TR = 2,180 ms, scan time 5:15 min; University of British Columbia—TE = 3.6 ms, TR = 1,858 ms, scan time 6:23 min. The testing “CIHR” data included 3D T1‐weighted images exclusively acquired at the University of Alberta (1.5 T Siemens Sonata) site using an MPRAGE sequence (1 × 1 × 1 mm^3^) with TE = 4.38 ms, TR = 1,870 ms, TI = 1,100 ms, flip angle = 15°, scan time 4:29 min. Other images were also acquired over 25 min included T2‐weighted, fluid‐attenuated inversion recovery, resting‐state functional (for NeuroDevNet), and DTI; however, none of these are the focus of the current report on brain volumes.

### 2.4 Automated brain segmentation

In this study, only regional brain volumes rather than other imaging metrics were used as predictors for classification because reductions in regional brain volumes have been the most commonly reported differences in FASD populations relative to controls (Donald et al., 2015; Lebel et al., 2011). Regional brain volumes were extracted from T1‐weighted structural images using the automated segmentation pipeline FreeSurfer version 5.3 (Fischl, 2012). Volumetric loss relating to FASD has been observed in numerous brain regions (Donald et al., 2015) with some regions being consistently reported including: regions of subcortical gray matter, total white matter, corpus callosum, and regions of the cortex. Hence, volumes of 87 regions were selected for classification analysis including subcortical gray matter (12–6 regions for left and right), left/right total white matter (two left and right), corpus callosum segmentations (five regions), and cortical parcellations (68–34 regions for left and right). Note that left and right segmentations were kept separate for analysis. Notably, ventricular segmentations were excluded based on limited reports of volumetric differences in FASD, right/left nucleus accumbens were excluded based on the low scan‐rescan reliability of FreeSurfer segmentations (Morey et al., 2010), and cerebellum/brain stem were excluded due to partial coverage in many participants. Each included volume was then standardized across training and test datasets (i.e., mean centered to zero and scaled to unit variance over entire training/testing datasets) as this is a requirement of the support vector learning algorithm used to build a classification model.

### 2.5 Predictive model training

Using the brain volumes from the training data as input, a linear support vector machine (SVM) was trained to predict FASD or control using the scikit‐learn machine learning toolbox version 0.18.1 (Pedregosa et al., 2011). This SVM algorithm was selected based on accurate performance in other neurological and psychiatric disease classification studies (Orrù, Pettersson‐Yeo, Marquand, Sartori, & Mechelli, 2012) and a linear kernel was used to allow for the identification of highly contributing brain regions to the model. The multisite data were selected for training so that the classification model generated by the SVM was robust to between site variation of regional brain volume measurements, and would perform consistently across different sites. A single classification model was generated by fitting the SVM hyperparameter “C” based on the training data using a combination of leave‐one‐out cross‐validation with internal 10‐fold validation for parameter selection. For each internal fold, the soft margin constant “C” was selected from a list of possible values (10^−4^, 10^−3^, 10^−2^, 1, 10, 100) as the parameter with the highest average accuracy over the 10‐fold internal validation. A single value of “C” for the training data was then chosen as the mode of all selected parameters from the leave‐one‐out folds and a single classification model was fit to the entire training data. This model was then used to predict FASD or control for each subject in the test data.

### 2.6 Model evaluation/interpretation

Three measures of model performance were calculated on both the leave‐one‐out cross‐validation training results and the test dataset predictions, namely, accuracy, sensitivity, and specificity. In addition, normalized feature weights (decision boundary weight divided by maximum weight in model) of the trained model were investigated to identify brain regions that contributed the most to FASD prediction. To compare the performance of the multivariate prediction model to more conventional univariate analysis, the same training/testing procedure was performed on each of the 87 individual brain volumes separately. Both cross‐validation training and test set accuracies were compared between the multivariate model and all other univariate models. Permutation tests were performed on multivariate and univariate test accuracies by calculating the accuracy of the trained models on 2,000 permutations of test data labels (FASD/Control). Note that because of the number of evaluations performed, only multivariate/univariate models that performed higher than a multiple comparison corrected *p*‐value (*p* < .0005 = .05/88 tests) on the testing data permutation tests are presented.

### 2.7 Sensitivity of model to participant demographics

To test for sensitivity of the classifier to FASD subgroup, the number of true positives (TPs) and false negatives (FNs) was compared between the three subtypes (FASD with sentinel facial features, FASD without sentinel facial features, and confirmed PAE without official FASD diagnosis). Next, the distance from support vector decision boundary was calculated for each subject in the test data as a measure of how closely a subject matched the FASD prediction model. A positive boundary distance value indicates the subject was predicted “control,” whereas a negative value indicates the subject was predicted “FASD.” For comparison between models, distance values were scaled by the maximum absolute distance of the test samples. Regional brain volumes are known to differ between males and females (Cahill, 2006; Cosgrove et al., 2007) and change throughout childhood/adolescence with regionally specific developmental trajectories (Giedd et al., 1999; Narvacan et al., 2017). Boundary distances were used to test for systematic classification errors related to sex (*t* test), age (linear regression), and age‐by‐sex interaction (linear regression). To test for sensitivity of the classifier to a specific cognitive phenotype, linear regression was performed between boundary distance and two separate behavioral tests which were Woodcock Johnson Quantitative Concepts (mathematics) and Woodcock Johnson Word Identification (reading), notably these were the only tests performed in a majority of individuals from both the training and testing cohorts. All statistical tests were performed separately for FASD and control groups and corrected for multiple comparisons (Bonferroni correction: five tests by two groups, 10 comparisons, effective *p* < .005).

### 2.8 Sex specific modeling

Following these primary analyses, two approaches were taken to address sex‐related differences in model performance. Approach 1: The addition of sex as a control variable in a linear regression is a common approach for addressing sex‐related variation in classification studies (some examples; Fair et al., 2013; Nielsen et al., 2013). In this study, the entire modeling procedure was repeated with brain volumes adjusted for sex using a linear regression prior to model training. Approach 2: The same modeling procedure was performed on raw brain volumes for males (*n* = 35 FASD, *n* = 32 controls) and females (*n* = 44 FASD, *n* = 49 controls) separately in the training cohort and then applied to the males (*n* = 38 FASD, *n* = 42 controls) and females (*n* = 29 FASD, *n* = 32 controls) in the test cohort. Both correction techniques were compared to the original model (which did not account for sex‐related variation) using measures of accuracy, sensitivity, and specificity separately for males and females in the test cohort.

## 3 RESULTS

### 3.1 FASD classification model/performance

**Figure 1:**
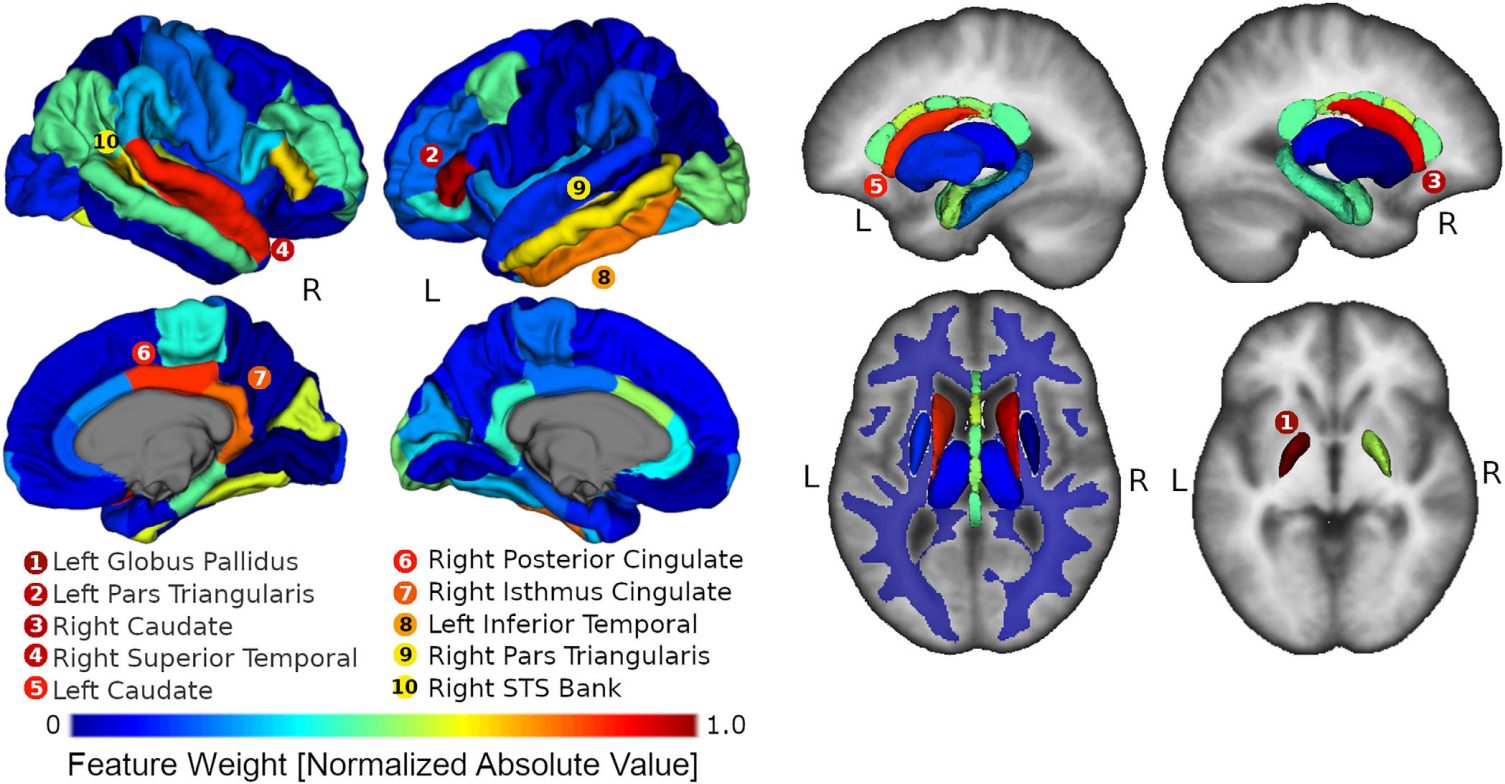
Visualization of model generated from brain volumes from the training dataset. All regions are displayed as 3D renderings, with the exception of left/right white matter segmentations displayed as a transparent overlay on an axial image from the FreeSurfer average template. For visualization purposes, globus pallidus renderings are displayed on a separate axial image. All regions of the brain are color coded by normalized support vector classifier weightings (weight divided by maximum absolute value weight in model). The 10 most heavily weighted regions are listed in order with corresponding colors. Model weightings were strongest for left/right regions of the temporal lobes, subcortical regions (left/right caudate and left globus pallidus), bilateral frontal lobe regions (left/right pars triangularis), and two regions in the cingulate of the right hemisphere

A binary classification model based on brain volumes was created to discriminate between typically developing individuals and those with FASD. The 10 most heavily weighted brain regions in the model included three subcortical gray matter regions (left globus pallidus, left and right caudate), three cortical gray matter regions located in the temporal lobe (right superior temporal gyrus, bank of the right superior temporal gyrus, and left inferior temporal gyrus), two cortical regions located in the frontal lobe (left and right pars triangularis), and two along the cingulate gyrus (right posterior cingulate and right isthmus of the cingulate). A visualization of all model weights for each segmented brain region is shown in Figure 1.

**Figure 2:**
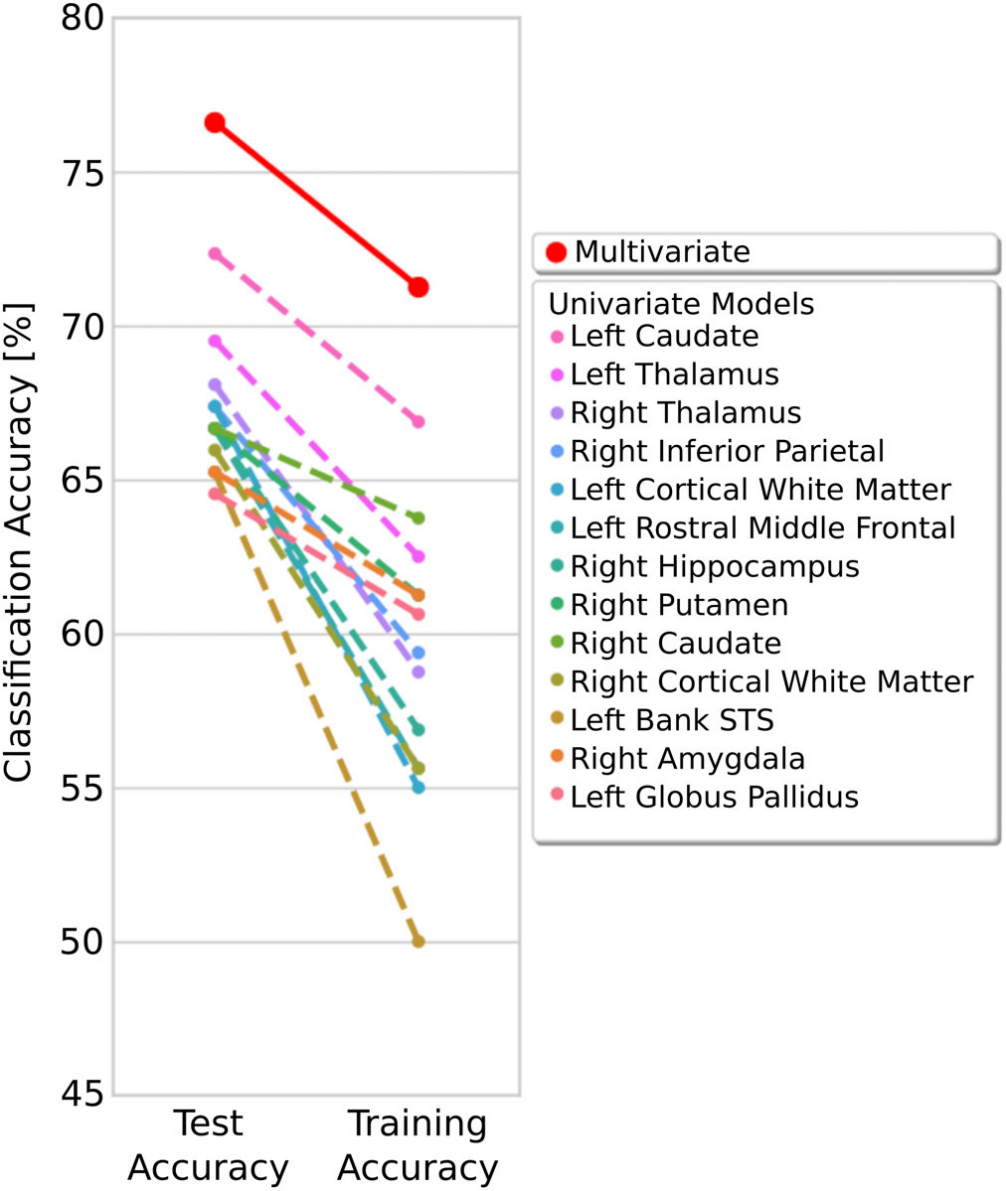
Performance of the multivariate brain volume prediction model (solid red line) compared to models generated using each brain region volume separately (dashed lines). Both the accuracy of the models on the test data and leave‐one‐out cross‐validation accuracy on the training data are shown. Models are listed from highest to lowest accuracy and are presented if they performed significantly greater than chance (permutation test, *p* < .0005) in the test cohort. The multivariate model outperformed all univariate models in both the training and testing data. Notably, 8 of these 13 regions are deep gray matter structures including bilateral caudate and bilateral thalamus

**Figure 3:**
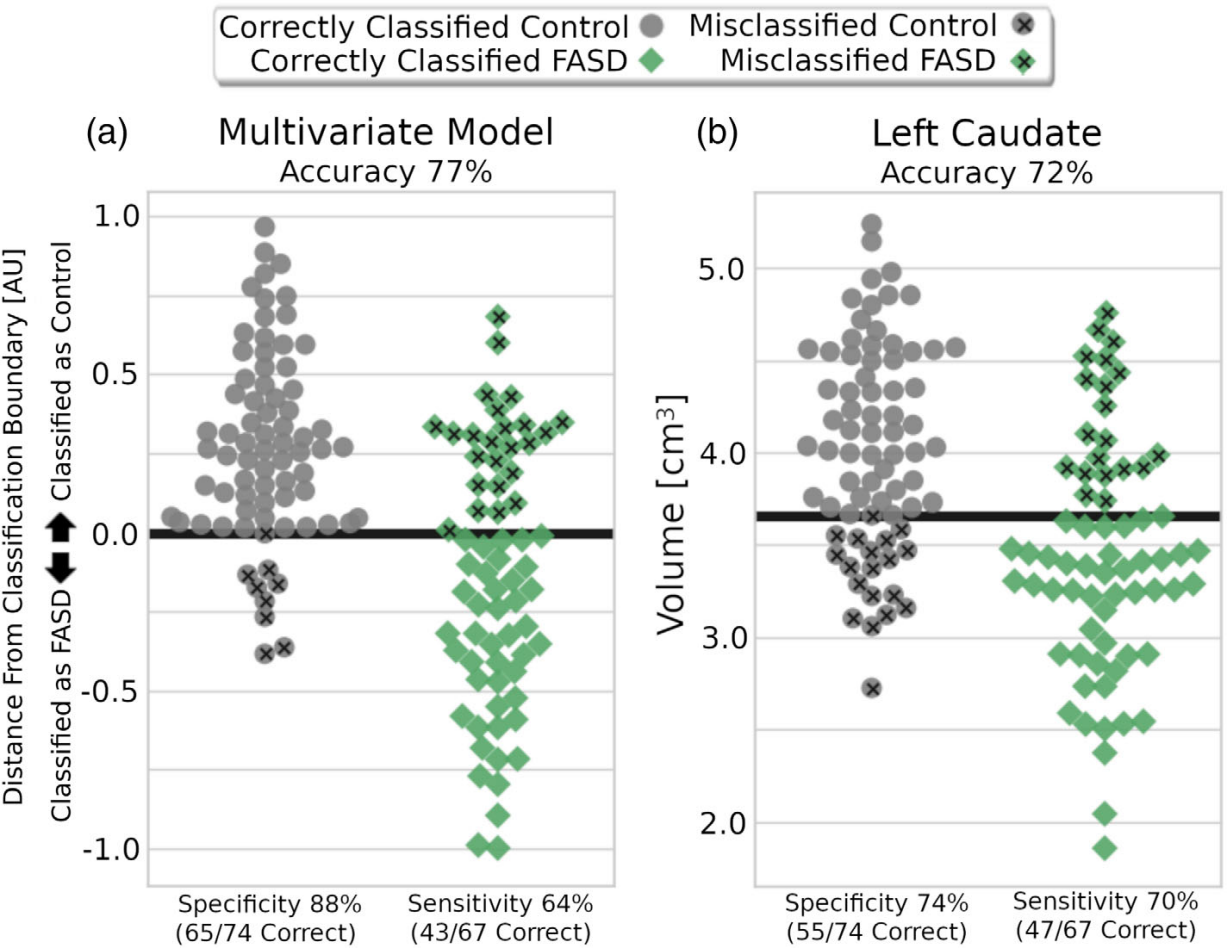
Distance from classification boundary for multivariate classification model (a) and raw volume for the top performing univariate model (b, left caudate) are presented for all subjects in the test “Canadian Institutes of Health Research” (CIHR) cohort separated by group (fetal alcohol spectrum disorder [FASD]/control). Values above the support vector machine (SVM) decision boundary (black line) were classified as the control group whereas values below the decision boundary were classified as FASD. A large proportion of FASD participants had a distance from decision boundary that did not overlap any of the controls (20 FASD with boundary distance <−0.38), and similarly a large proportion of FASD had minimal overlap with controls below a left caudate volume of ~3.1 cm^3^ (1 control, 17 FASD)

The model showed moderate performance on the training data (NeuroDevNet) with accuracy 71%, sensitivity 58%, and specificity 84%, and achieved similar results on the independent test data (CIHR) with accuracy 77% (*p*‐value = .0005), sensitivity 64%, and specificity 88%. Notably, the multivariate classification model outperformed all univariate classification models for accuracy in the test data (77% compared to the next highest 72% for the left caudate model), and in the training data (71% compared to 67% for the left caudate model). Accuracies for the multivariate and univariate classification models are presented in Figure 2. The multivariate classification model was more specific (88%) compared to all other univariate models that achieved higher than chance classification accuracy (58% right hippocampus–85% right putamen), whereas sensitivity of the multivariate model (64%) was within range of the above chance univariate models (right putamen 46%–79% left thalamus). Boundary distance, accuracy, sensitivity, and specificity for the multivariate classification model are presented alongside the left caudate univariate model in Figure 3. Notably in both the univariate left caudate volume and multivariate model distance measurements, there was a significant proportion of FASD participants minimally overlapping the controls. In total, 17 FASD participants (10 females; age 12.22 ± 3.43 years; seven FASD with sentinel facial features, four without sentinel facial features, and six prenatally exposed without official diagnosis) had a left caudate volume smaller than ~3.1 cm^3^ whereas only one control had a left caudate volume below that threshold. No controls and eight FASD participants had a left caudate volume lower than ~2.7 cm^3^. Similarly, the multivariate model had 20 FASD participants (nine females; age 12.23 ± 3.27 years; seven FASD with sentinel facial features, six FASD without sentinel facial features, and seven prenatally exposed without official diagnosis) with no overlapping controls below a distance from decision boundary value of −0.38 (the lowest control value). Of these, 20 FASD participants that did not overlap controls in the multivariate model, five had a caudate volume larger than 3.1 cm^3^ demonstrating that the multivariate and univariate models are discriminating different individuals.

### 3.2 Diagnostic, demographic, and cognitive associations to FASD classification model

When separating classification performance in the FASD group by the three diagnostic subtypes, differences were observed between the proportions of TPs to FNs between subtypes. Notably, almost all the FASD subjects with sentinel facial features were correctly classified (11 TPs, 3 FNs), whereas the other two subtypes, FASD without sentinel facial features (21 TPs, 14 FNs) and PAE (11 TPs, 7 FNs), had a lower proportion of TP relative to FN.

T tests revealed a systematic difference in classification boundary distance between control males and females (*t*‐statistic −3.67, *p*‐value = .0005) indicating more false positives for females compared to males which may be a result of lower brain volumes observed in typical females relative to typical males, as a group. Linear regression results relating classification boundary distance to four demographic variables of interest, namely, age, age by sex, WRMT‐R Word Identification (reading), and Woodcock Johnson Quantitative Concepts (mathematics) were not significant in the FASD or control groups.

### 3.3 Sex specific models

To further investigate the effect of sex on model performance, the original classification model was evaluated separately for males and females. The classification accuracies of the entire training set were similar for males (76%, *p* = .0005) and females (77%, *p* = .0005), but sensitivity was lower and specificity was greater for males (sensitivity 53%, specificity 98%) compared to females (sensitivity 79%, specificity 75%). In other words, 1/42 male controls were misclassified as FASD, whereas 8/32 female controls were misclassified as FASD. A larger difference in classification accuracy was observed in the FASD groups where 17/38 male FASD were misclassified as controls whereas only 6/29 female FASD were misclassified.

**Figure 4:**
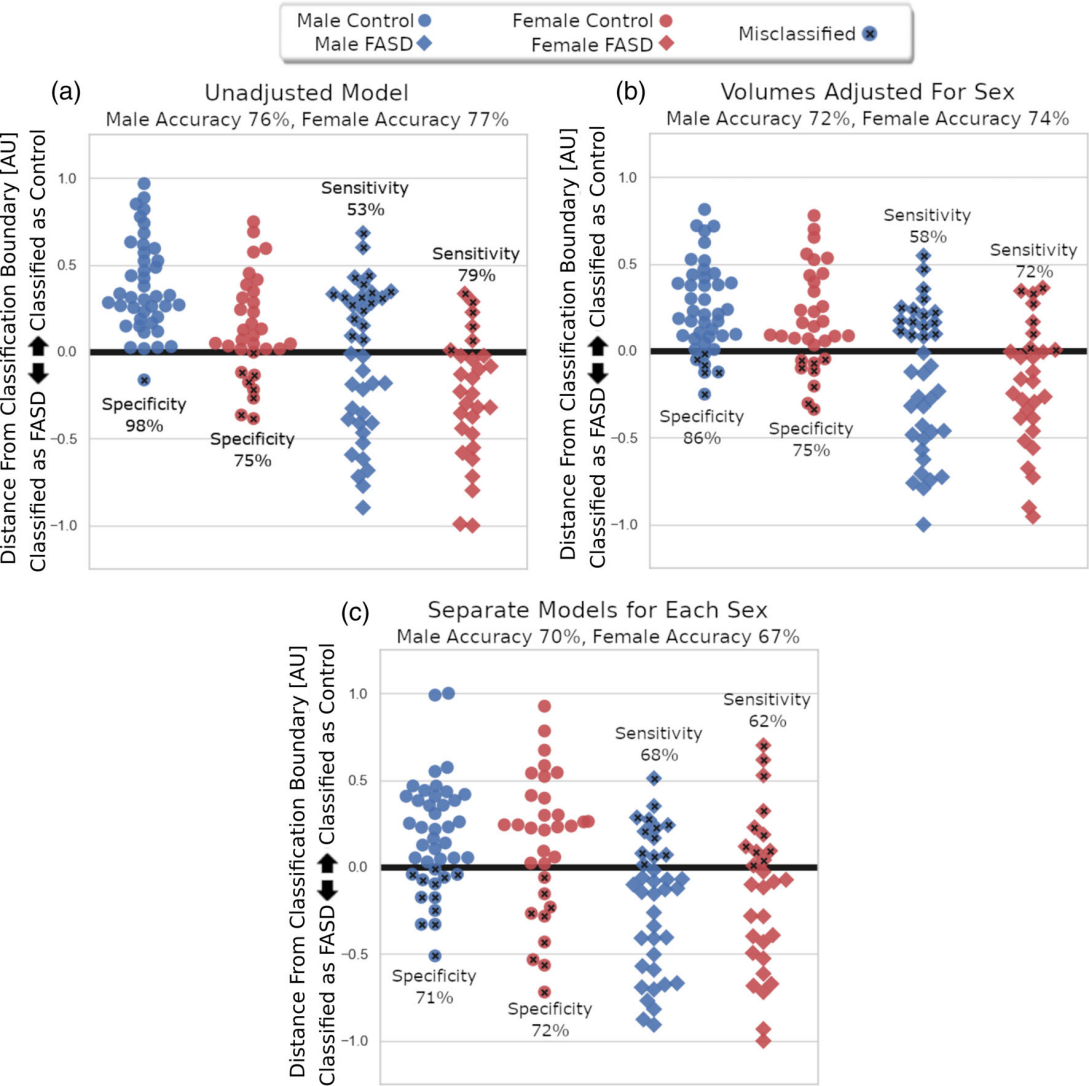
Distance from classification boundary is presented here for each subject in the test data separated by group (control, circle/fetal alcohol spectrum disorder [FASD], diamond) and sex (male, blue/female, red) for the unadjusted multivariate model (a), the multivariate model using regional brain volumes adjusted for sex (b), and creating separate classification models for males and females (c). Positive values indicate the subject was classified to the control group while negative values indicate the subject was classified to the FASD group. The most misclassifications in the unadjusted model were male FASD participants labeled as controls (18/38 misclassified) and a notable number of female controls were incorrectly labeled FASD (8/32 misclassified). Adjusting brain volumes for sex improved imbalance in specificity between males compared to females, whereas creating separate models improved the sex‐related imbalance in both specificity and sensitivity

The first approach for reducing sex‐related bias in sensitivity/specificity was to fit a model based on sex adjusted volumes. This approach performed moderately well on the test data; however, sensitivity remained low for males relative to females: male accuracy 72% (*p*‐value = .0005), sensitivity 58%, specificity 86%; female accuracy 74% (*p*‐value = .0005), sensitivity 72%, specificity 75%. The second approach for reducing sex‐related bias in FASD prediction was to create separate models for males and females. Both models performed moderately well on the test data and had similar sensitivity and specificity between males and females: male accuracy 70% (*p*‐value = .0005), sensitivity 68%, and specificity 71%; female accuracy 67% (*p*‐value = .01), sensitivity 62%, and specificity 72%. Notably, sensitivity in the male FASD group was increased by creating separate models for males and females at the cost of decreased specificity and overall accuracy. Classification performance and classifier boundary distance separated by sex and group is presented in Figure 4 for the original model, the model adjusted for sex and the models created separately for each sex.

**Figure 5:**
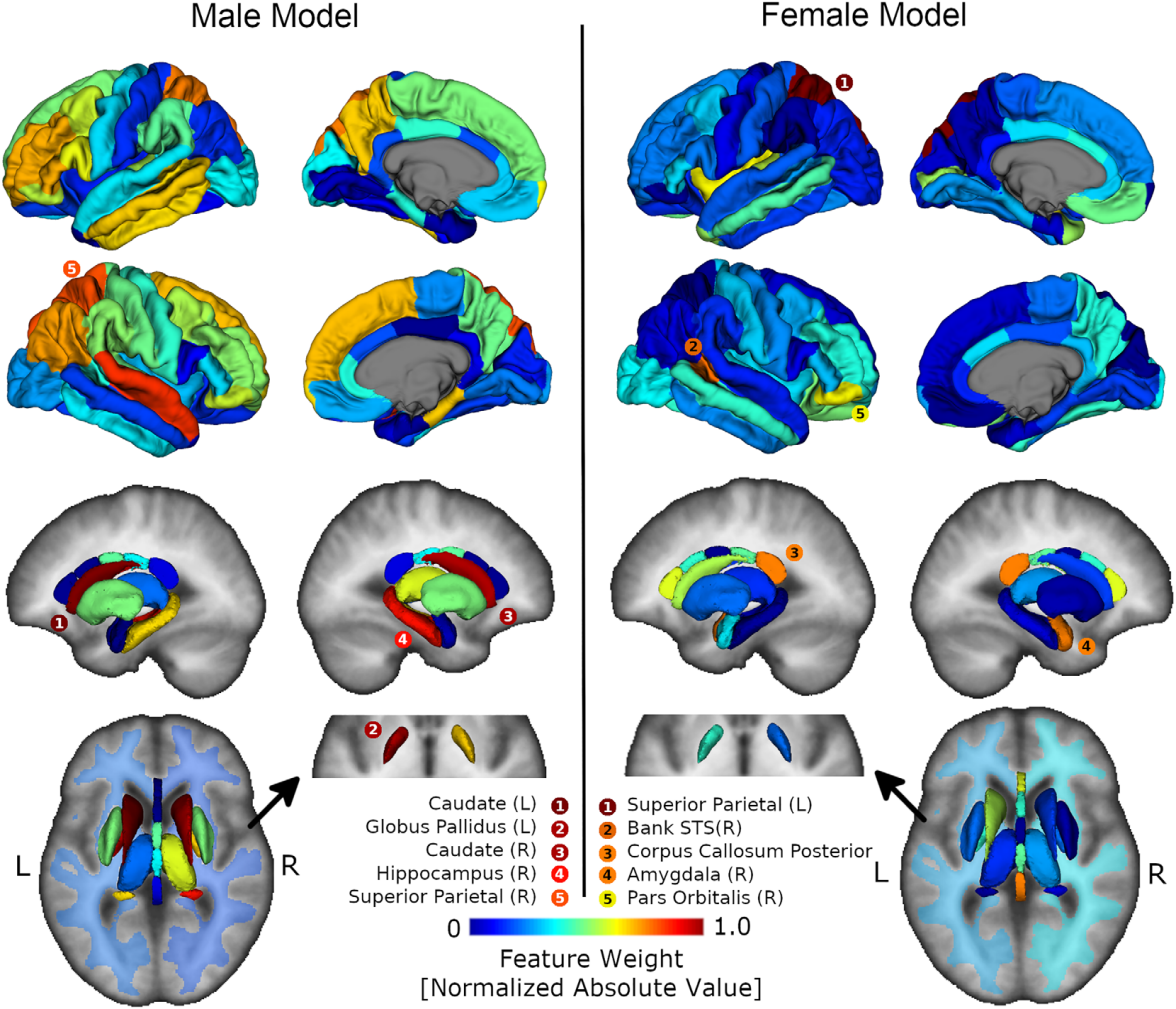
Visualization of prediction models generated on the training data for males (32 controls, 35 fetal alcohol spectrum disorder [FASD]) and females (49 controls, 44 FASD) separately. All regions are displayed as 3D renderings, with the exception of left/right white matter segmentations displayed as a transparent overlay on an axial image. All regions of the brain are color coded by support vector classifier weightings (feature importance). The five most heavily weighted regions in each model are listed in order with corresponding colors. Four of the five most heavily weighted regions in the male model are subcortical structures whereas four of the five most heavily weighted regions in the female model are cortical or corpus callosum regions

In general, the predictive models created separately for males and females had primary contributions from volumes of different brain regions. Of the five most heavily weighted regions in the male model, four were subcortical gray matter regions (left globus pallidus, left/right caudate, right hippocampus), and one was the right superior parietal region of the cortex. In contrast, of the 5 most heavily weighted regions in the female model, only one was subcortical gray matter (right amygdala), three were cortical regions (left superior parietal, right bank of the superior temporal gyrus, and right pars orbitalis), and one was the posterior part of the corpus callosum. The model weights for the male and female models are visualized in Figure 5.

## 4 DISCUSSION

### 4.1 FASD classification

This study reports a multivariate classification model based on brain volume that showed moderate accuracy (77% in test cohort) in identifying individuals with FASD from control participants. Notably, this classification accuracy is comparable to that reported in neuroimaging classification studies of other neurodevelopmental disorders such as ADHD (81% accuracy using resting state functional MRI measurements, Fair et al., 2013) and Autism (70% accuracy using brain volume and resting state functional MRI measurements, Zhou et al., 2014). To our knowledge, only one other study that primarily focused on eye tracking/psychometric data has attempted classification of FASD participants from controls using imaging data. In that study, an accuracy of 67% was achieved using features extracted from diffusion MRI of the corpus callosum on a relatively small sample (training: 19 controls, 11 FASD, testing: 22 controls, 24 FASD) of children/adolescents that partly overlapped the training cohort from the current study (also from NeuroDevNet) (Zhang et al., 2019). Notably, the accuracy using diffusion MRI features extracted from the corpus callosum was lower than the accuracy reported from the current study using brain volumes (77%); however, the same study reported highest accuracies using features derived from other physiological/behavioral measurements (e.g., eye tracking data 76% and psychometric data 78%).

Other studies have classified FASD participants based on other modalities such as epigenetic DNA methylation features where a predictive model trained on an overlapping cohort from the training dataset in the current study achieved 83% accuracy in predicting FASD (Lussier et al., 2018). Additionally, features extracted from 3D facial laser scans achieved ~80–90% accuracy identifying individuals with FAS (Fang et al., 2008), a subtype of FASD that exhibits sentinel facial features, the same subtype of FASD that had a high classification accuracy with multivariate brain volumes in the current study (11/14 FASD participants with sentinel facial features correctly classified in the test cohort). In a three‐way classification task of FASD, ADHD and control participants, a 77% classification accuracy was achieved using features extracted from eye tracking data collected while participants attended to videos (Tseng et al., 2013).

Taken together, these studies suggest that there may be value in combining multiple types of diagnostic and clinical features in future classification models to improve accuracy, including other modalities of MR imaging such as diffusion MRI and resting state functional MRI which were not part of this current analysis on brain volumes. Multimodal classification of FASD has been performed using features derived from psychometric and eye tracking data achieving 83% accuracy (Zhang et al., 2019), but showed no additional accuracy when including diffusion MRI; however, the sample size was limited in that study (22 controls, 24 FASD).

Changes in total brain volume as well as unique regional trajectories of subcortical and cortical gray matter development during childhood/adolescence (Giedd et al., 1999; Narvacan et al., 2017) may impact classifier performance. In a supplementary analysis of classification performance (data not shown), no difference in age was observed between incorrectly/correctly classified controls or between incorrectly/correctly classified individuals with FASD in the test cohort, suggesting that classification performance was not confounded by age.

### 4.2 Relating multivariate and univariate analysis of FASD regional brain volumes

In this study, the multivariate FASD classification model outperformed all univariate models that were based on separate brain region volumes by ~5% in both the test and training cohorts. This result suggests that there is a pattern of volume change involving multiple brain structures that are more discriminative of children/adolescents with FASD relative to any one brain region independently. Of the univariate models with above chance accuracy, regions are consistent with previous studies reporting volume loss associated with FASD (Donald et al., 2015). Above chance univariate models consisted of eight subcortical gray matter structures (left/right caudate, left/right thalamus, right putamen, right hippocampus, right amygdala, left globus pallidus), both left/right white matter volumes, and three cortical gray matter regions (right inferior parietal, left rostral middle frontal gyrus, left bank of the superior temporal sulcus). On the other hand, multivariate model weights indicated fewer subcortical regions as heavily contributing to prediction (left/right caudate and left globus pallidus in the top 10), whereas cortical regions in the temporal lobe (left inferior temporal, bank of the right superior temporal sulcus, right superior temporal sulcus) and other subdivisions of the cortex (right posterior cingulate, right isthmus cingulate, right/ left pars triangularis) were more prominent. Taken together, univariate and multivariate results suggest that the increased accuracy of the multivariate model (relative to univariate models) may be a result of the inclusion of cortical regions.

In the current study, a model trained using only the left caudate had a test accuracy only 5% lower than the model generated from all brain regions together. Notably, the caudate was one of the first reported brain structures with differences associated with PAE (Mattson et al., 1996). Since then, the caudate has been reported in animal models to be one of the more vulnerable regions to ethanol‐induced apoptosis (Young & Olney, 2006) which may underlie the observed volume reductions associated with prenatal exposure to alcohol in children and adolescents (Astley et al., 2009; Cortese et al., 2006; Riikonen et al., 2005). Additionally, caudate volume has also been associated with deficits in both cognitive control and verbal learning/recall in children/adolescents with FASD (Fryer et al., 2012). In the current study, the classification model based solely on left caudate volume outperformed (72% test accuracy) the models based on other basal ganglia structures (e.g., left globus pallidus [64% accuracy], left putamen [63% accuracy], right putamen [67% accuracy], right caudate [67%]). The caudate takes input from the frontal eye fields and the frontal/parietal lobes, and has efferent pathways to the prefrontal cortex. Other basal ganglia structures such as the putamen and globus pallidus are implicated in neural pathways related to motor function. Hence, a larger effect of PAE on the caudate relative to other basal ganglia structures may reflect larger deficits in FASD to higher order cognitive functions (e.g., executive function, problem solving) compared to motor functions. Additionally, a more recent study has demonstrated that shape‐based features of caudate asymmetry can be combined with facial morphology features to better discriminate controls from those with FAS (Suttie et al., 2018). Taken together, these findings suggest that the caudate is one of the most heavily impacted brain structures post‐PAE. In this study, a left caudate volume decision boundary of ~3.6 cm^3^ (larger size indicating control) was generated from the training data and performed adequately (accuracy 72%; sensitivity 70%; specificity 74%) on the test data. Notably, a large proportion of FASD participants (17/67) had left caudate volumes lower than all but one control at a threshold of 3.1 cm^3^ and suggests that a volume threshold could be used as a highly specific indicator of FASD. Similarly, distance from the decision boundary of the multivariate model is highly specific at values lower than −0.38 with 20/67 FASD participants and no controls having values below this threshold. Interestingly, at these lower values both left caudate volume and distance from the multivariate decision boundary were not sensitive to a particular sex or FASD diagnostic subtype, suggesting that both these measures may provide added value for further subdividing the FASD diagnosis based on brain structure alone. Importantly, the FASD model performed more accurately in the subtype of FASD with sentinel facial features relative to the other participants that did not display these features, suggesting a pattern of FASD brain volume change that is also likely to be associated with other structural changes in an individual. This finding is consistent with other univariate studies showing that facial dysmorphic features are related to more severe volumetric reductions in FASD (Astley et al., 2009; Roussotte et al., 2012), and may reflect the timing of ethanol exposure between 3 and 4 weeks postgestation in humans when the brain and face are early in their development (Godin et al., 2010; Godin, Dehart, Parnell, O'Leary‐Moore, & Sulik, 2011). Notably, more extensive volumetric reductions in the dysmorphic FASD participants could also be related to a higher level of prenatal ethanol exposure (although this was unavailable in our study) complicating the face–brain interpretation.

Along with ADHD, the participants in this study had a wide range of comorbid diagnoses (e.g., ADHD, oppositional defiant disorder, etc.). Importantly, to be of clear clinical use, a classification model would be able to discriminate individuals with FASD from those with other neurodevelopmental disorders. Results from this study demonstrate that individuals with FASD can be discriminated from controls using regional brain volumes. However, it is unknown whether regional brain volumes or the same classification model could be used to discriminate individuals with FASD from those with other neurodevelopmental disorders.

The investigation of model weights can also aid in identifying regions that may be affected in FASD but that are not detected by univariate analysis alone. In this study, both the left and right pars triangularis of the frontal lobe heavily contributed to the model. Notably, the volume of the bilateral pars triangularis has been associated with reading disorders such as dyslexia (Eckert et al., 2003) and deficits in language have been repeatedly observed in complex language tasks in participants with FASD (Becker, Warr‐Leeper, & Leeper, 1990; Mattson, Riley, Gramling, Delis, & Jones, 1998). Although the frontal lobe has shown volume loss in children/adolescents prenatally exposed to alcohol, to our knowledge pars triangularis volume has not been associated with FASD. Given that the pars triangularis regions were absent from the univariate models that performed higher than chance, this result implies that in the context of other FASD‐related regional volume change a multivariate model can extract additional information about structural change that is undetectable by univariate analysis alone.

### 4.3 FASD classification with sex specific models

To date, the most common approach for dealing with sex‐related variation in large classification studies of neurodevelopmental disorders is to perform classification on volumes adjusted for sex resulting from a multivariable linear regression with sex added as a covariate (some examples being: Fair et al., 2013; Zhou et al., 2014). However, in neurodevelopmental disorders such as FASD where reductions in regional brain volumes appear to be larger for males relative to females (Chen et al., 2012;Dudek et al., 2014 ; Treit et al., 2017) assuming the same effect of sex on volume between controls and FASD will have the effect of reducing but not eliminating between sex bias in sensitivity/specificity. Results from the current study demonstrate experimentally that when sex is not accounted for in FASD classification, sensitivity/specificity can differ greatly for males (sensitivity 53%, specificity 98%) compared to females (sensitivity 79%, specificity 75%) but this disparity can be reduced at the expense of accuracy by using sex adjusted volumes (male accuracy 72%, sensitivity 58%, specificity 86%; female accuracy 74%, sensitivity 72%, specificity 75%). Furthermore, this study proposes building FASD classification models separately for males and females which further reduced the imbalance in sensitivity/specificity, albeit at a larger decrease in accuracy (male accuracy 70%, sensitivity 68%, and specificity 71%; female accuracy 67%, sensitivity 62%, and specificity 72%). The observed decrease in accuracy of separate male/female models may be a result of the limited sample size for males/females in the training data and would likely be improved with the inclusion of more participants. An advantage of this technique is the ability to investigate heavily contributing regions that are useful for prediction of FASD in males and females separately. Notably, subcortical regions heavily contributed to male FASD prediction (left/right caudate, left globus pallidus, right hippocampus) whereas cortical regions primarily contributed to female FASD prediction (left superior parietal cortex, right bank of the superior temporal gyrus, right pars triangularis), suggesting that patterns of volume change differ between males and females. The higher subcortical weightings in the male classification model likely reflects the greater relative volume reductions of subcortical gray matter regions in males compared to females with FASD (Dudek et al., 2014; Treit et al., 2017). Several neurophysiological/neurochemical effects of PAE are reported to be greater in males relative to females, including reductions in long‐term potentiation (Sickmann et al., 2014), increases in dopamine D1R binding (Converse et al., 2014), and reduced sensitivity to testosterone (Lan, Hellemans, Ellis, Viau, & Weinberg, 2009). More heavily weighted cortical regions in the female model is surprising, given that previous studies have reported no significant differences in the volume of cortical regions in females with FASD (Chen et al., 2012) and less pronounced effects of PAE on measures of cortical thickness relative to subcortical volume (Treit et al., 2017). It seems here that a pattern (i.e., multivariate) of cortical volume reduction may more accurately discriminate females with FASD from controls compared to PAE related volume change within individual (i.e., univariate) cortical regions. Overall, results from this study suggest that there is value in modeling FASD related regional brain volume change separately for males and females. Notably, the classification differences reported here between males/females could be confounded by sex by group imbalances in demographics. However, no such group by sex interaction effects were observed in the test cohort for any of the demographic variables listed in Table 2 (data not shown), suggesting that demographic imbalances are not driving the observed classification differences between males and females. In the training cohort, a small difference in age was observed between male control (age 11.3 ± 3.5 years) and male FASD (age 13.3 ± 2.7 years) participants, potentially impacting the weightings of the male FASD classification model. However, this male classification model heavily weighs subcortical regions whose volumes have been shown to change minimally over childhood/adolescence in both longitudinal and cross‐sectional samples (Narvacan et al., 2017), suggesting that age differences are not influencing the model weightings presented in this study.

### 4.4 Study limitations and future directions

There are several limitations in this study, primarily related to the imbalanced distribution in demographics/comorbidities in the training/testing FASD groups relative to controls. The samples in this study consisted of control groups primarily of Caucasian descent, whereas about half the FASD participants self‐identified as indigenous potentially confounding classification results. However, in a follow‐up analysis, sensitivity to FASD classification differed minimally between the ethnic categories in the testing cohort (indigenous: 63%; Caucasian 67%; other 60%; unknown 64%) suggesting that ethnicity was not influencing classification performance. ADHD is a common comorbid diagnosis within FASD populations having an estimated prevalence of >70% (Burd, Klug, Martsolf, & Kerbeshian, 2003) and was highly prevalent in the training/testing cohorts included in the current study (training FASD: 50%; testing FASD: 49%). Additionally, in this study, a large proportion of FASD participants were on medication regimens that were highly discordant between individuals, and those participants were not asked to refrain from taking medication throughout the study. Such confounds in comorbid diagnosis and medications may impact reported cognitive scores and classification results in the FASD group. Again, a secondary analysis was conducted and showed minor differences between classification sensitivity between an ADHD‐comorbid diagnosis (67% sensitivity) /no‐ADHD diagnosis (62% sensitivity), as well as classification of FASD participants on different medications (stimulants 58%, atypical antipsychotics 59%, antidepressants 60%, and other medication 67%). This equally distributed sensitivity among demographic categories suggests that even though the FASD classification model was generated from imbalanced control/FASD training data, the model itself represents a discriminative pattern of brain volume difference that is associated with PAE and does not reflect differences based on ethnicity, comorbid diagnosis or medication regimen.

The training and testing FASD cohorts of the current study contained both individuals with a formal FASD diagnosis as well as those with confirmed alcohol exposure but nondiagnosed. Importantly, the classification results from the test cohort showed similar sensitivity between the FASD participants without sentinel facial features (test sensitivity 60%), and those in the PAE (nondiagnosed) group (test sensitivity 61%), suggesting that regional brain volumes were similarly affected in the diagnosed and undiagnosed individuals. In a secondary analysis excluding the PAE group (data not shown), decreased accuracy and sensitivity was observed in the test cohort (accuracy 74%, sensitivity 53%, specificity 88%) relative to when PAE (nondiagnosed) were included (accuracy 77%, sensitivity 64%, and specificity 88%) warranting the inclusion of the PAE group in the analysis.

## 5 CONCLUSIONS

In this study, a multivariate classification model was generated for discriminating children/adolescent controls from those with FASD. The model performed better than univariate analysis in discriminating FASD from controls and had predictive contributions from regions with known volumetric reduction in FASD. Additionally, a large proportion of FASD participants in the test data had little to no overlap with controls at negative distance from boundary values, and low left caudate volume values, suggesting that these measures should be investigated as a potential indicator of FASD. Classification accuracy of models generated separately for males and females had lower accuracy than the model containing all participants, but notably these models were more balanced in sensitivity and specificity suggesting that sex should be taken into account in brain volume‐based classification of FASD. Overall, this study shows the value in multivariate analysis of brain volume for the classification of FASD and identification of brain regions affected in children and adolescents prenatally exposed to alcohol.

## Data Availability

Research data are not available.
